# Why Does Child Mortality Decrease With Age? Modeling the Age-Associated Decrease in Mortality Rate Using WHO Metadata From 25 Countries

**DOI:** 10.3389/fped.2021.657298

**Published:** 2021-08-11

**Authors:** Josef Dolejs, Helena Homolková

**Affiliations:** ^1^Department of Informatics and Quantitative Methods, University of Hradec Králové, Hradec Králové, Czechia; ^2^Division of Pediatric Neurosurgery, Department of Pediatric and Trauma Surgery, Thomayer's Teaching Hospital and Third Faculty of Medicine, Charles University in Prague, Prague, Czechia

**Keywords:** mortality rate, age, childhood, congenital anomalies, WHO database

## Abstract

**Background:** Our previous study analyzed the age trajectory of mortality (ATM) in 14 European countries, while this study aimed at investigating ATM in other continents and in countries with a higher level of mortality. Data from 11 Non-European countries were used.

**Methods:** The number of deaths was extracted from the WHO mortality database. The Halley method was used to calculate the mortality rates in all possible calendar years and all countries combined. This method enables us to combine more countries and more calendar years in one hypothetical population.

**Results:** The age trajectory of total mortality (ATTM) and also ATM due to specific groups of diseases were very similar in the 11 non-European countries and in the 14 European countries. The level of mortality did not affect the main results found in European countries. The inverse proportion was valid for ATTM in non-European countries with two exceptions.

Slower or no mortality decrease with age was detected in the first year of life, while the inverse proportion model was valid for the age range (1, 10) years in most of the main chapters of ICD10.

**Conclusions:** The decrease in child mortality with age may be explained as the result of the depletion of individuals with congenital impairment. The majority of deaths up to the age of 10 years were related to congenital impairments, and the decrease in child mortality rate with age was a demonstration of population heterogeneity. The congenital impairments were latent and may cause death even if no congenital impairment was detected.

## Introduction

Human mortality rate decreases with age after birth and increases with age in adults. This increase in adults is exponential and it is usually interpreted as a manifestation of aging and affects all individuals (1–23). Age is a deterministic variable in the relationship because coefficients of determination were higher than 0.99 (if age as a single independent variable is used to explain the changes of mortality rate, then more than 99% of variability is explained) (4, 19–22). All historical changes in healthcare such as reduction of mortality due to infectious diseases or due to cardiovascular diseases did not affect the shape of the relationship in the last two centuries and they changed only two parameters of the model (11, 12, 18, 23).

Faster changes in mortality rate with age occur during childhood (5, 8, 9, 20–28). The steep mortality decrease is accompanied by age-based changes in the causes of death (28). For example, congenital anomalies and impairments originating in the perinatal period reached more than 85% of all deaths during the first 4 weeks of life and only 10% in the age 5–10 years interval in 14 European countries [furthermore, “the age 5–10 interval” corresponded to the mathematical interval (5, 10)] years and to the age category 5–9 years in demography) (28). Simultaneously, the decrease of total mortality rate was smooth and was described by the model of the inverse proportion with a coefficient of determination higher than 0.99 (20–28).

The presented paper closely follows the previous study (28). Age trajectory of total mortality (ATTM) was more important than age trajectories of mortality due to specific diseases and the decrease of ATTM was described by the model of the inverse proportion up 10 years in the previous study (28).

With respect to the whole world, 14 European countries studied in the previous study came from a homogenous region (e.g., with the respect to the health system or economy level). This study aimed at investigating the previous results in other continents and in countries with a higher level of child mortality; data from 11 non-European countries were used.

## Materials and Methods

The description of age trajectory of mortality (ATM) during the first year of life was essential to observed results, and the WHO mortality database was used. For these reasons, the selection of countries and calendar years in the database was based on two criteria:

a) The ICD10 classification was used.
b) Four age categories were used in the first year of life (it was the maximal number of age categories used in the database in the first year).

Unfortunately, the list of countries that comply to the second criterion was not big. For example, India, China, Russia, or African countries did not meet it. Finally, 11 countries were selected in the database.

Age was assumed as the main factor, and all other factors were assumed to be less significant (28). Furthermore, the existence of general mechanisms was assumed, as demonstrated in the ATM after birth. For these reasons, ATM was primarily constructed in as large a population as possible. Including more regions and calendar years within the analysis may eliminate all factors other than age, rendering the impact of age more visible. The same methods that were used in the previous study were used to calculate the mortality rate (28). The unit corresponding to the mortality rate was “person-years,” which was the number of years lived by members of the population between age limits of age category.

### Aggregated Populations

Zero deaths in a given age group may make it impossible to construct ATM for some set of diseases. While zero deaths due to some disease may occur within a specific age category in a specific calendar year, at least one death may occur within the same age category in another calendar year. Consequently, the inclusion of additional calendar years and regions may remove this obstacle. The aggregation of more calendar years was first utilized by Edmond Halley in 1693 (29, 30). The method enables the calculation of the mortality rate within one age category based on the number of deaths and living persons in several years and more regions (28–31). Besides, it may be assumed that ATM may be smoother in an aggregated population according to the law of large numbers.

The WHO mortality database contains the number of deaths Di within specific age categories in different countries (32). The database uses the following four age categories for the first year of life: “0 days” [(0, 24) hours], “1–6 days” [(1, 7) days], “7–27 days” [(7, 28) days], and “28–365 days” [(28, 365) days]. One or two age categories are also used in the database in the first year and such data were not convenient. Cause of death was determined using the specific revision of the International Classification of Diseases in the database. Here, the calendar period used in each country corresponds to the period when the 10th revision (ICD10) and the four age categories were applied (33). The numbers of living people are obtained from the U.S. Census Bureau (Bureau of the Census 2019) (34).

The present study used data collected in the following 11 non-European countries: Argentina, Brazil, Peru, Venezuela, Chile, Colombia, Mexico, Japan, Australia, New Zealand, and the USA. The first six countries were aggregated to population P4 (South America) while the last five countries were aggregated to population P5. Besides, aggregated populations P1, P2, P3, and P14 were defined and used in the previous study (28). Finally, the data from 11 non-European countries and 14 European countries from the previous study were aggregated to the largest population P25 (28). The sum of the means of population sizes calculated in a specific country in a specific calendar period was 1,306,115,570 living persons per one calendar year (it may be interpreted as the average size of P25). Population P25 was about 3.3 times bigger than P14 and represented a significant portion of the world population. Calendar periods and means of population sizes of populations are shown in the second and in the third columns in Table 1 (28). If no deaths were registered within a specific age category due to specific diseases in any population, ATM for this population and set of diseases was not constructed. For example, no cases in the chapter “Pregnancy, childbirth, and the puerperium” (XV) were found within the age range (0, 10) years in all populations. Thus, this chapter was not relevant to the study.

**Table 1:** Population sizes and results of total mortality calculated during the first 10 years in all populations in the log-log scale.

**Population**	**Years**	**Size**	**Slope γ**	**Lower**	**Upper**	**level μ_1_**	**${\bar{R}}^{2}$**	**$R_{\text{b}}^{2}$**	**c/x**
**Part A: Previous study**									
France	2000–2014	61,466,098	−0.984	−1.03	−0.93	58.0	0.9963	0.9965	0.47
Germany	1998–2015	80,892,654	−0.987	−1.04	−0.94	54.5	0.9964	0.9967	0.54
Italy	2003–2014	59,026,383	−1.007	−1.07	−0.94	49.3	0.9941	0.9948	0.81
Spain	1999–2015	44,137,863	−0.960	−1.02	−0.90	58.3	0.9941	0.9931	0.17
UK	2001–2015	61,569,167	−1.030	−1.08	−0.98	60.0	0.9963	0.9959	0.22
**P1**	x	307,092,165	−0.997	−1.05	−0.95	56.6	0.9965	0.9970	0.87
Czech Republic	1994–2015	10,384,837	−0.918	−1.01	−0.83	70.2	0.9873	0.9810	0.06
Austria	2002–2016	8,420,447	−1.006	−1.07	−0.94	52.0	0.9944	0.9951	0.82
Hungary	1996–2015	10,055,552	−1.008	−1.08	−0.94	90.8	0.9937	0.9945	0.79
Poland	1999–2015	38,570,112	−1.023	−1.09	−0.96	79.9	0.9946	0.9948	0.41
Slovakia	1996–2014	5,407,663	−0.951	−1.03	−0.87	77.6	0.9901	0.9887	0.19
**P2**	x	72,838,611	−1.003	−1.06	−0.94	77.5	0.9948	0.9955	0.92
Sweden	1997–2015	9,215,809	−0.968	−1.02	−0.91	46.5	0.9953	0.9948	0.22
Norway	1996–2015	4,708,433	−0.966	−1.01	−0.92	54.6	0.9967	0.9959	0.13
Denmark	1994–2009	5,357,073	−1.014	−1.06	−0.97	48.1	0.9970	0.9972	0.51
Finland	1996–2015	5,265,968	−0.960	−1.03	−0.89	50.9	0.9919	0.9913	0.23
**P3**	x	24,547,283	−0.975	−1.03	−0.92	49.8	0.9960	0.9959	0.30
**P14**	x	404,478,059	−0.996	−1.05	−0.95	60.1	0.9962	0.9967	0.88
**Part B: Present study**									
**P14**	x	404,478,059	−0.996	−1.05	−0.95	60.1	0.9962	0.9967	0.88
Argentina	2003–2014*	40,916,085	−1.020	−1.09	−0.95	156.2	**0.9923**	0.9928	0.55
Brazil	2006–2015	196,482,134	−1.034	−1.11	−0.96	165.6	0.9933	0.9931	0.30
Peru	1999–2015*	28,032,803	−0.917	**−0.98**	−0.85	187.4	0.9929	0.9857	**0.02**
Venezuela	1996–2013	26,147,140	−0.992	−1.08	−0.90	224.1	0.9885	0.9899	0.85
Chile	1997–2015	16,109,643	−1.012	−1.07	−0.95	111.4	0.9947	0.9952	0.66
Colombia	1997–2013*	42,606,241	−0.971	−1.03	−0.91	183.5	0.9945	0.9943	0.29
**P4**	x	350,294,046	−1.007	−1.08	−0.94	174.6	0.9935	0.9943	0.80
Mexico	1998–2012*	108,139,515	−1.002	−1.07	−0.93	192.9	0.9930	0.9939	0.94
Japan	1995–2015	126,809,629	−0.871	**−0.92**	−0.82	57.1	0.9948	0.9735	**0.00**
Australia	1998–2015	20,616,599	−1.003	−1.07	−0.94	61.4	0.9943	0.9950	0.92
New Zealand	2000–2013	4,094,611	−0.985	−1.06	−0.91	79.3	0.9911	0.9920	0.66
USA	1999–2010*	291,683,111	−1.010	−1.10	−0.93	89.4	0.9903	0.9914	0.78
**P5**	x	551,343,465	−0.994	−1.05	−0.94	102.2	0.9959	0.9964	0.81
**P25**	x	1,306,115,570	−1.002	−1.06	−0.95	111.7	0.9961	0.9965	0.92

Size population is mean of population sizes in specific calendar years. Data was not collected in the whole calendar interval labeled as “*” in the second column; exact calendar years were: in Argentina:2003, 2005–2014, in Peru:1999–2000, 2002–2008, 2011–2015, in Colombia:1997–1998, 2010–2013, in Mexico:1998, 2000, 2001, 2007, 2008, 2010, 2011, 2012 and in USA:1999–2005, 2009–2010. The column labeled as “slope γ” contains point estimation of the slope in the linear two parametric model in the log-log scale and the next two columns (“Lower” and “Upper”) contain the limits of 95% confidence intervals of the parameter γ. The parameter μ_1_ is per 100,000 living per 1 year and was calculated in the two parametrical linear model. ${\bar{R}}^{2}$ is the adjusted coefficient of determination calculated for one predictor and nine points in the linear two parametrical model. $R_{\text{b}}^{2}$ is the coefficient of determination calculated for the inverse proportion with a single parameter in the log-log scale (it was automatically also the adjusted coefficient of determination) (28). The last column labeled as “c/x” contains p-value of the standard Fisher's test, which determined that the linear model with two parameters does not provide a significantly better fit than the inverse proportion with a single parameter (which may slowly differ from the values of μ_1_ calculated in the two parametrical linear model).

Sums Di of death numbers from diseases of all main ICD10 chapters, within all populations and within all age categories, and sums Li of living individuals within specific age categories are in the file “25_Countries_Dolejs_Homolkova” Animations are in eight files in “mp4” format and other results are in the file “Appendix” The files may be found in the Supplementary Material.

The level of statistical significance was set to 0.05 for all tests. The arithmetic mean of the interval endpoints was used as a representative value for each age category. The time unit “1 year” was used in all age categories and in all calculations.

### Chapters of the ICD10

Cause of death may be considered less reliable information than the determination of age. On the other hand, ATM from specific diseases may show the composition of ATTM and may show some other important results (e.g., extraordinary different shape of ATM due to neoplasms) (26, 28).

Two chapters of ICD10 “Diseases of the eye and adnexa” (VII) and “Pregnancy, childbirth, and the puerperium” (XV) were not relevant to the study because only 10 cases were in chapter VII and zero cases were detected in chapter XV in the largest population P25 in the whole age interval (0, 10) years. ATM in all other chapters of the ICD10 and ATM due to congenital anomalies of the central nervous system (CACNS) were constructed. CACNS were the subset of the chapter “Congenital malformations, deformations, and chromosomal abnormalities” (XVII), and ATM due to CACNS was constructed because it showed a higher coefficient of determination up to higher ages in the previous studies (26–28).

Diseases from chapters for which the decrease in mortality was slower during the first year than after the first year were not related to congenital impairment were aggregated to the group labeled “Other diseases.” This group contained chapters I–XV, without chapter II (Neoplasms) (28).

### Data Processing and Statistical Methods

Data processing, statistical methods, and model assessment were the same as in the previous studies (24, 27, 28). A simple software was developed using Visual Basic for Applications, and Microsoft (MS) Excel 2019 was used for the calculation of the sum of cases in each chapter and each age category. MS Excel was used at the first level of processing, along with more packages in R 4.0.2 for Windows for statistical calculations (linear and non-linear regression) and preparation of charts. The level of statistical significance was set to 0.05 for all tests. Overview of other possible models and more detailed description of model assessment may be found in previous studies (24, 27, 28).

## Results

### Age Trajectory of Total Mortality

ATTM was more important and had a more general meaning than other ATMs that were constructed in specific groups of diseases. Besides, the determination of the cause of death may be burdened with some uncertainty (for example, it may be expected for cases registered in the 18th chapter of the ICD10: “Symptoms, signs and abnormal clinical and laboratory findings, not elsewhere classified”).

ATTM had reached the minimal value in all 32 studied populations (in 25 countries and 7 aggregated populations) in the age interval 5–10 years with three exceptions (the minimal value was in the next age interval 10–15 years in Slovakia, Peru, and Japan). All ATTMs were statistically evaluated in the age interval 5–10 years in all 32 populations, and the results calculated in the log–log scale are shown in Table 1. Table 1 contains all populations from the previous study (14 countries and aggregated populations P1, P2, P3, and P14) and all populations from the presented study (11 countries and the aggregated populations P4, P5, and P25) (28).

### Inverse Proportion Assessment in the Log–Log Scale

**Figure 1:**
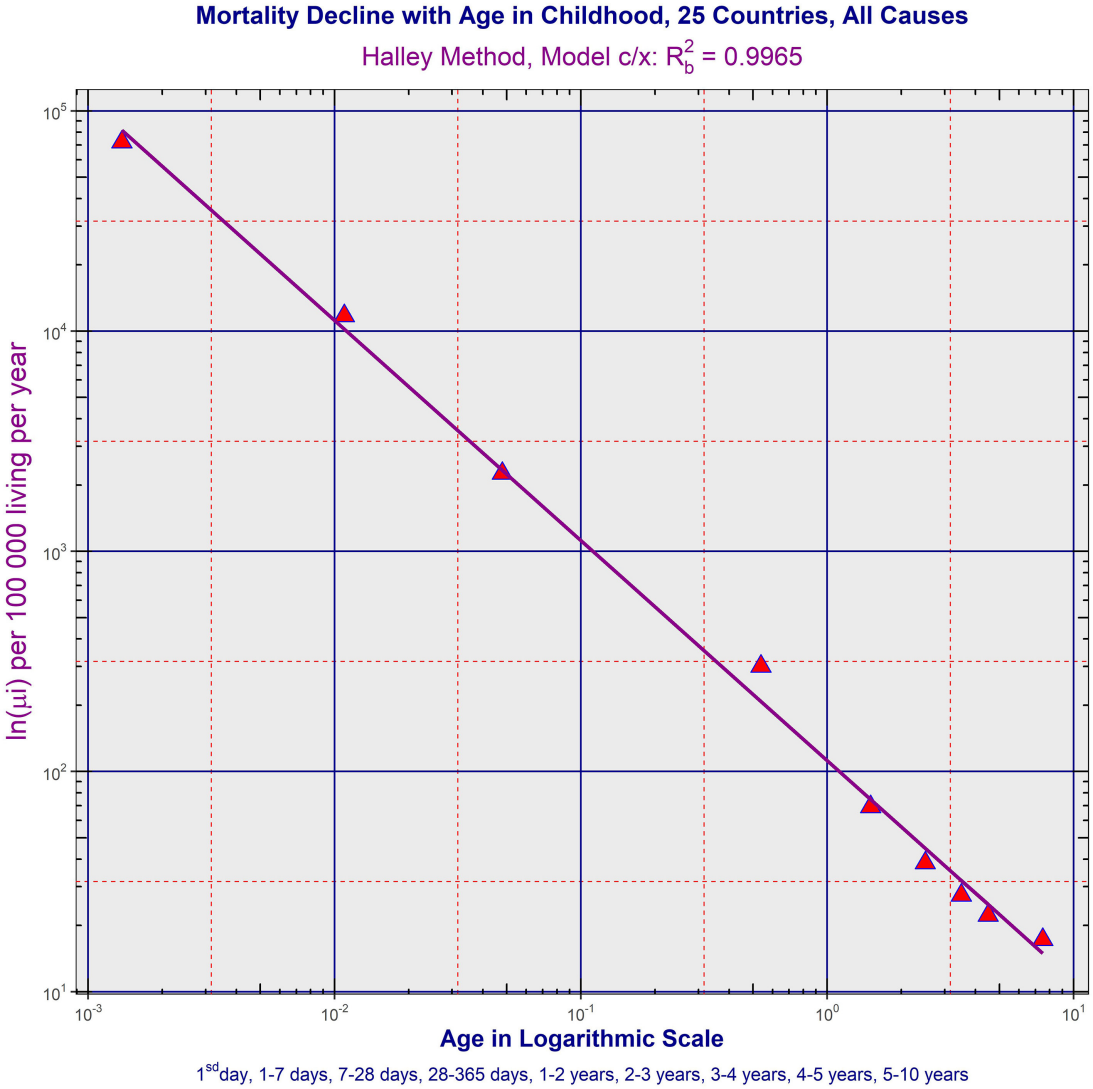
Age trajectory of total mortality in P25. (All_Populations_All_causes_Animation_1.mp4).

The decrease of ATTM was visually linear in the log–log scale. It is shown in Figure 1 for P25 and for all populations in the file “All_Populations_All_causes_Animation_1.mp4” presented in the Supplementary Material. The linearity in the log–log scale was tested in the quadratic model using the method of least squares (LS). The null hypothesis that the quadratic element was zero was not rejected (*p* > 0.05), while the linear element was significant (*p* < 0.0001) in all populations (the results are not shown in Table 1). Consequently, it was assumed that the decrease in total mortality with age was linear in the log–log scale. At the second step, a linear model with two parameters was developed in the log–log scale (27, 28). The parameters were ln[μ_1_], γ, where γ is the slope of the straight line in the log–log scale. The two parameters, their standard deviations, and the adjusted coefficients of determination *R*^2^ were calculated using the LS method for the age interval of 0–10 years. The hypothesis that the residuals were age-independent was not rejected (*p* > 0.05). The results of the linear regression are shown in Table 1.

The specific value −1 for the slope **γ** corresponded to the inverse proportion between mortality rate and age. Because the slopes **γ** were close to the value −1 in the fourth column in Table 1, the null hypothesis H_o_: **γ** = −1 was examined to test the inverse proportion at the last step (all formulas were described in detail in the previous study) (28). The inverse proportion with a single parameter was a nested model that includes the two-parameter linear model. The null hypothesis that the model with two parameters did not provide a significantly better fit than the inverse proportion was tested using a standard Fisher's test. The resulting *p*-values are shown in Table 1 in the last column (the null hypothesis was rejected only in Peru and Japan). Because the slopes are more or less close to −1, the inverse proportion between total mortality and age may be the resulting model within the age range of 0–10 years.

### Chapters of the ICD10

**Figure 2:**
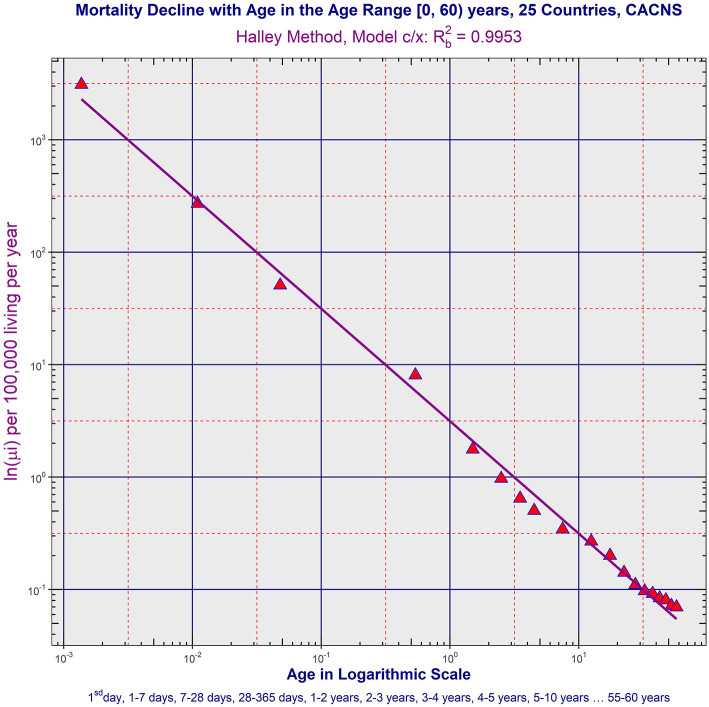
Age trajectory of CACNS in P25. (All_Populations_CACNS_Animation_2.mp4).

**Figure 3:**
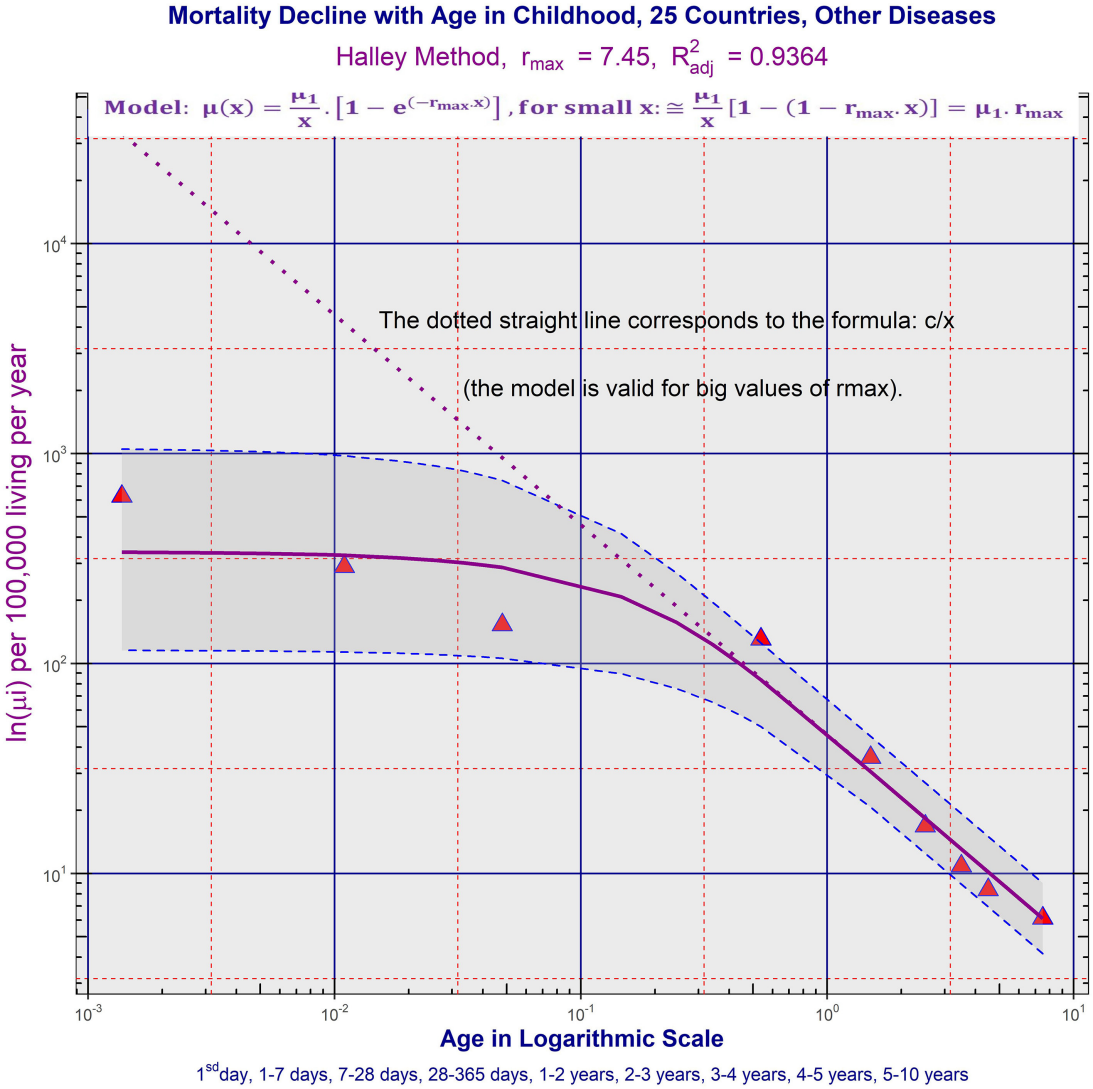
Age trajectory of “Other diseases” in P25. (All_Populations_Other_diseases_Animation_3.mp4).

Three main results described in the previous study were confirmed for non-European populations from three other continents and/or with higher mortality levels (all files mentioned below may be found in the Supplementary Material):

a) ATM due to CACNS decreased according to the inverse proportion up to higher ages. The model of inverse proportion was not rejected in all aggregated populations and in 12 countries in the age range (0, 15) years (it was the maximal age range where ATM due to CACNS was constructed). Besides, mortality minimum was reached in the age category (55, 60) years in the largest population P25 with ${\text{R}}_{\text{b}}^{2}$ = 0.9953. ATM due to CACNS in P25 is shown in Figure 2, and ATM due to CACNS in all 32 populations is shown in the file “All_Populations_CACNS_Animation_2.mp4.” More detailed results calculated in ATM due to CACNS in all populations are in Table 2, and other notes are in the file “Appendix.”
b) ATMs due to the majority of chapters of ICD10 were age-independent or slowly decreased during the first year, and simultaneously, these ATMs decreased according to the inverse proportion in the age range (1, 10) years. ATM due to the specific category “Other diseases” is shown in Figure 3 in P25. ATMs due to “Other diseases” in all 32 populations are shown in the file “All_Populations_Other_diseases_Animation_3.mp4,” ATMs due to the first chapter “Certain infectious and parasitic diseases” of ICD10 are shown in file “All_Populations_Chapter_1_Animation_4.mp4,” and ATMs due to all chapters of ICD10 in P25 and P14 are shown in the file “All_Chapters_P25_and_P14_Animation_5.mp4.” TCIR explained child mortality decrease with age based on the sequential extinction of more severe impairments. It also explained the bending ATM using formula (2), which was derived in previous studies (25–28). If the maximal congenital individual risk of death r_max_ in the born population is not big, then Equation (1) is valid:
  $$\begin{array}{l} \mu \left(x\right)=\frac{\mu_{1}}{x}.\left[1-e^{\left(-r_{max}.x\right)}\right],\text{and for small} \end{array}$$
  (1) $$\begin{array}{l} x:≅\frac{\mu_{1}}{x}\left[1-\left(1-r_{max}.x\right)\right]=\mu_{1}.r_{max} \end{array}$$
More detailed results calculated in ATM due to all chapters of ICD10 in population P25 are in Table 3 and other notes are in the file “Appendix.”
c) The shapes of ATM due to neoplasms were very different. It was confirmed that ATMs due to neoplasms were age-independent in all populations after the first year of life (the independence of age was tested in the age range 1–10 years as in other chapters of ICD10 and also in the age range 1 month−15 years. Simultaneously, the ATM due to neoplasms decreased in the first month of life (the model of inverse proportion was rejected only in P1, Argentina and Venezuela where the decrease was slightly slower). ATM due to neoplasms in all populations is shown in the file “All_Populations_Neoplasms_Animation_6.mp4.” More detailed results calculated in ATM due to neoplasms in all populations are in Table 4, and other notes are in the file “Appendix.”

## Discussion

According to the theory of congenital individual risks of death (TCIR), the mortality decrease was explained as the result of depletion of individuals with more severe congenital impairments (25, 26, 28). The inverse proportion was explained in TCIR as the result of the specific distribution of congenital impairments in the born population. The spectrum of congenital anomalies was very wide because some individuals died during the first hours and others may survive to higher ages. The born population was considered as a whole and the changes of individual congenital risks of death were assumed as age-independent (possible individual changes of individual risk of death were assumed to be negligible with respect to differences in the whole population) (25, 26, 28). The evidence that empirical ATTM decreased according to the inverse proportion was interpreted by the following rule: “as more severe the impairment was than less frequent it was in born population” (25). The rule may be caused by selection in previous generations or by selection in the prenatal period (25, 26, 28).

The empirical decrease of ATMs was very fast in European countries after birth, and the absolute value of the decrease to 10 years was approximately the same as the absolute value of the increase with age in the age range 10–90 years (28). ATMs after birth were studied in the log–log scale where the assumptions of the regression model were satisfied. The model of inverse proportion is a formally linear model with the slope −1 in the log–log scale. The empirical coefficients of determination calculated in the inverse proportion in ATTM were very high (>0.99) (26–28). ATM due to congenital anomalies of the central nervous system (CACNS) decreased according to the model of inverse proportion over the age of 50 years and the coefficients of determination were higher than 0.99 (27, 28). The decrease of ATM with the slope −1 in the log–log scale was also observed in other groups of diseases in the age range 1–10 years while the decrease was slower during the first year of life (these ATMs were labeled “bending ATMs”) (26, 28).

The inverse proportion was valid for ATTM in non-European countries with the two exceptions (Peru and Japan). Such high values of coefficient of determination ${\text{R}}_{\text{b}}^{2}$ in Table 1 are uncommon, and the inverse proportion between total mortality and age was a deterministic relationship. It implies that, compared to the first day of life, the mortality rate was 10 times lower after 10 days, 100 times lower after 100 days, and 3,650 times lower after 10 years.

Because the adjusted coefficients of determination calculated for ATTM in the two parametric linear model for R^2^ were high in Japan and Peru (0.9948 in Japan and 0.9929 in Peru), the lower value of ${\text{R}}_{\text{b}}^{2}$ was due to slopes that differ from the value −1. ATTMs in Peru and Japan were also exceptional with respect to the age category of the minimal mortality value (the minimal value of ATTM was reached in the age category 10–15 years in the two populations). The explanation of the results is difficult without any speculation. One possibility is that the slower decrease was related to the Japanese population, which is known for high life expectancy. The effect was less significant in Peru (with a steeper decrease than in Japan), and simultaneously, the population in Peru contains a subpopulation of Japanese (Japan Peruvians), which constitute ~1.4% of the population of Peru. The speculation may show that the slower mortality decrease may be exclusive to the Japanese population. Another possibility is that the quality of data collection in Japan and Peru may be another reason for the non-conformance of the inverse proportion model. This may be true for one or both countries (35, 36). On the other hand, the determination of the age of death represents more reliable information. The used age categories were relatively wide and possible uncertainty may be found rather in the determination of the cause of death.

ATMs due to CACNS differed from other diseases because they reached the minimal value in higher ages, and the inverse proportion model was valid with high coefficients of determination (26–28). The age range 0–15 years was used here to compare ATM from CACNS as, in all populations studied, this was the lowest age range in which mortality decreased. All 32 ATMs due to CACNS were available in the age range 0–15 years. Curvature was not rejected in nine countries (Germany, Italy, Austria, Poland, Slovakia, Finland, Australia, New Zealand, and the USA), while it was rejected in all other countries and in all aggregated populations where the linear model was assumed. In the next step, the inverse proportion was tested in the remaining 16 countries and all 7 aggregated populations (in ATMs due to CACNS where the curvature was rejected). The inverse proportion was rejected only in France, Czech Republic, Hungary, and Brazil. Consequently, the inverse proportion was confirmed in 12 countries and in all aggregated populations in the age range (0, 15) years. The coefficients of determination calculated in the model of inverse proportion ${\text{R}}_{\text{b}}^{2}$ were very high, in general reaching the maximum value of 0.9942 in the largest population P25, and the value of 0.9937 in P14. All results are shown in Table 2 in Appendix. The coefficients of determination ${\text{R}}_{\text{b}}^{2}$ calculated for ATM from CACNS were almost as high as those calculated for total mortality (Table 1 in the main text). For example, the coefficient of determination was 0.9965, for total mortality within the age range (0, 10) years in P25, while a value of 0.9938 for ATM from CACNS was found in P25 in the same age range. ATM from CACNS decreased up to 60 years in the largest population P25, and the coefficient of determination ${\text{R}}_{\text{b}}^{2}$ reached 0.9953 in the age range (0, 60) years in P25. The ATM is shown in Figure 2 and all other ATMs due to CACNS are shown in the file “All_Populations_CACNS_Animation_2.mp4” in the Supplementary Material.

Results observed in ATM due to specific diseases also support TCIR. If TCIR is correct, a significant part of the deaths up to 10 years was, in fact, caused by congenital impairment, even in the cases registered in the category “Other diseases.” Figure 4 in the file “Appendix” illustrates age changes of proportions of deaths from diseases categorized in specific chapters of ICD10. Because ATM due to the majority of chapters decreased according to the inverse proportion after the first year, the deaths may have been caused by latent congenital impairment. For example, 23% of the deaths until the age of 10 years were due to conditions grouped in the category “Other diseases” in P25. Simultaneously, ATM due to “Other diseases” was bent according to Equation (2).

ATM due to neoplasms differed from other diseases. The evidence that ATMs due to neoplasms decreased in the first month of life and were age-independent within the age range (1 month, 15 years) may be important for the verification of any hypothesis that a specific type of neoplasm is only significant to a subpopulation with congenital predisposition, for example, a genetically susceptible subpopulation.

### Quality of WHO Data and Death Classification

The determination of cause of death according to ICD may be related to some dissimilarity in different countries (35–40). For example, mortality data from 27 countries of the WHO European region were analyzed for coronary heart disease. Comparisons of mortality rates from coronary heart disease as reported in national mortality registries between countries and over time were highly compromised (35). The studies also described the regional differences in death classification (35–40). It may be partially followed in the animations “All_Populations_Chapter_1_Animation_4.mp4,” “All_Populations_Certain_conditions_in_perinatal_period_Animation_7.mp4,” and “All_Populations_CA_Animation_8.mp4,” which were presented in the Supplementary Material. Besides, bending ATMs were explained in TCIR by the fact that congenital defects were not observed in some cases, and these cases were not categorized as congenital anomalies. Two categories, CACNS and “Diseases of the central nervous system,” and ATM calculated in the categories represented a typical illustration of the effect, and the two ATMs may be found in the animation “All_Chapters_P25_and_P14_Animation_5.mp4” constructed in two populations P14 and P25. The regional differences in death classification may also be followed in the aggregated group of diseases “Other diseases.” It was created here for death cases that were not related to congenital impairment, malignant neoplasms, and accidents. The mortality level and shape of ATM due to “Other diseases” are shown in the animation “All_Populations_Other_diseases_Animation_3.mp4.”

### Consequences in Clinical Practice

According to TCIR and ATM constructed in 25 studied populations, the majority of deaths up to the age of 10 years may be related to congenital impairment, and the decrease in child mortality rate with age was a demonstration of population heterogeneity. The explanation of the mortality decrease with age leads to the existence of latent congenital defects. Such latent congenital defects may cause death even if no congenital impairment was observed and the cases were aggregated here in the group “Other diseases.” For example, death cases put in the first chapter of ICD10 “Certain infectious and parasitic diseases” may be associated with a congenital defect. Namely, if influenza was determined as a cause of death, then latent congenital impairment may be the dominant cause of death. Some other epidemiological studies also support the idea that a significantly higher proportion of individuals with an inherited predisposition are among these cases (41). These findings showed that there were two basic children's patients in clinical practice. The bigger group of patients was without congenital impairment and the smaller group was affected by congenital impairment. The heterogeneity in death cases was different (42, 43). The majority of death cases may be related to congenital impairment, which was latent. In other words, the group of patients with congenital impairment was relatively small in the whole population, but it may be dominant among dead up to the age of 10 years. More details of these findings may be found in the Appendix.

## Conclusion

ATTM and also ATM due to specific groups of diseases were very similar in 11 non-European countries and in 14 European countries. The level of mortality did not affect the main results found in 14 European countries.

Child mortality decrease with age may be explained as the result of the depletion of individuals with congenital impairment. The majority of deaths up to the age of 10 years were related to congenital impairments and the decrease in child mortality rate with age was a demonstration of population heterogeneity. The congenital impairments were latent and may cause death even if no congenital impairment was detected.

All results are based on published data, and the data are presented as a supplement in the file Table 1. It contains the numbers of living people in each age category, and each population is shown in Table 1. Besides, it contains the numbers of deaths in each age category, in each ICD10 chapter, and in each population aggregated in a calendar period. Consequently, more epidemiological findings may be done using simple calculations and the data.

## Data Availability Statement

The original contributions presented in the study are included in the article/Supplementary Materials, further inquiries can be directed to the corresponding author/s.

## Author Contributions

JD realized statistical computations and composed the main text. HH checked all parts related to pediatrics and clinical interpretations. Both authors contributed to the article and approved the submitted version.

## Conflict of Interest

The authors declare that the research was conducted in the absence of any commercial or financial relationships that could be construed as a potential conflict of interest.

## Publisher's Note

All claims expressed in this article are solely those of the authors and do not necessarily represent those of their affiliated organizations, or those of the publisher, the editors and the reviewers. Any product that may be evaluated in this article, or claim that may be made by its manufacturer, is not guaranteed or endorsed by the publisher.

## References

[1] Gompertz B. On the nature of the function expressive of the law of human mortality. Philos Trans R Soc London. (1825) 115:513–85. 10.1098/rstl.1825.0026

[2] Makeham W. On the law of mortality and the construction of annuity tables. J Inst Actuar. (1860) 8:301–10. 10.1017/S204616580000126X

[3] Heligman L Pollard JH. The age pattern of mortality. J Inst Actuar. (1980) 107:49–75. 10.1017/S0020268100040257

[4] Riggs JE. Longitudinal Gompertzian analysis of adult mortality in the US, 1900–1986. Mech Ageing Dev. (1992) 54:235–47. 10.1016/0047-6374(90)90053-I 2214892

[5] Luder HU. Onset of human aging estimated from hazard functions associated with various causes of death. Mech Ageing Dev. (1993) 67:247–59. 10.1016/0047-6374(93)90003-A 8326747

[6] Willemse WJ Koppelaar H. Knowledge elicitation of gompertz' law of mortality. Scand Actuar J. (2000) 2:168–79. 10.1080/034612300750066845

[7] Preston SH Heuveline P Guillot M. Demography: Measuring and Modeling Population Processes. Oxford: Blackwell (2001) 190–94 p.

[8] Bebbington M Lai CD Zitikis R. Modeling human mortality using mixtures of bathtub shaped failure distributions. J Theor Biol. (2007) 245:528–38. 10.1016/j.jtbi.2006.11.011 17188716

[9] Bebbington M Lai CD Zitikis R. Modelling deceleration in senescent mortality. Math Popul Stud. (2011) 18:18–37. 10.1080/08898480.2011.540173

[10] Harman D. Aging: a theory based on free radical and radiation chemistry. J Gerontol. (1956) 11:298–300. 10.1093/geronj/11.3.298 13332224

[11] Strehler BL Mildvan AS. General theory of mortality and aging. Science. (1960) 132:14–21. 10.1126/science.132.3418.14 13835176

[12] Hayflick L Moorhead PS. The serial cultivation of human diploid cell strains. Exp Cell Res. (1961) 25:585–621. 10.1016/0014-4827(61)90192-6 13905658

[13] Bjorksten J Tenhu H. The crosslinking theory of aging–added evidence. Exp Gerontol. (1990) 25:91–5. 10.1016/0531-5565(90)90039-5 2115005

[14] Lin XS Liu X. Markov aging process and phase type law of mortality. North Am Actuarial J. (2007) 11:92–109. 10.1080/10920277.2007.10597486

[15] Flores I Cayuela ML Blasco MA. Effects of telomerase and telomere length on epidermal stem cell behavior. Science. (2005) 309:1253–6. 10.1126/science.1115025 16037417

[16] Ferron SR Marques-Torrejon MA Mira H Flores I Taylor K Blasco MA. Telomere shortening in neural stem cells disrupts neuronal differentiation and neuritogenesis. J Neurosci. (2009) 29:14394–407. 10.1523/JNEUROSCI.3836-09.2009 19923274 PMC6665809

[17] Taupin P. Aging and neurogenesis, a lesion from Alzheimer's disease. Aging Dis. (2010) 1:89–104. 22396863 PMC3295028

[18] Zheng H Yang Y Land KC. Heterogeneity in the strehler mildvan general theory of mortality and aging. Demography. (2011) 48:267–90. 10.1007/s13524-011-0013-8 21347805

[19] Dolejs J. The extension of Gompertz law's validity. Mech Ageing Dev. (1998) 99:233–44. 10.1016/S0047-6374(97)00104-8 9483495

[20] Dolejs J Maresova P. Onset of mortality increase with age and age trajectories of mortality from all diseases in the four nordic countries. Clin Interv Aging. (2017) 12:161–73. 10.2147/CIA.S119327 28176929 PMC5268335

[21] Dolejs J. Modelling human mortality from all diseases in the five most populated countries of the European Union. Bull Mathematic Biol. (2017) 79:2558–98. 10.1007/s11538-017-0341-y 28887745

[22] Dolejs J. Age trajectories of mortality from all diseases in the six most populated countries of the south america during the last decades. Bull Mathematic Biol. (2014) 76:2144–74. 10.1007/s11538-014-0005-0 25124764

[23] Izsak J Juhász-Nagy P. On the explanation of the changes in age of the concentration of the death causes. Zeitschrift für Alternsforschung. (1984) 39:31–6. 6711018

[24] Dolejs J. Single parameter of inverse proportion between mortality and age could determine all mortality indicators in the first year of life. J Theor Biol. (2016) 397:193–8. 10.1016/j.jtbi.2016.03.007 26987522

[25] Dolejs J. Theory of the age dependence of mortality from congenital defects. Mech Ageing Dev. (2001) 122:1865–85. 10.1016/S0047-6374(01)00325-6 11557286

[26] Dolejs J. Analysis of mortality decline along with age and latent congenital defects. Mech Ageing Dev. (2003) 124:679–96. 10.1016/S0047-6374(03)00063-0 12735907

[27] Dolejs J Homolkova H Maresova P. Modeling the age-associated decrease in mortality rate for congenital anomalies of the central nervous system using WHO metadata from nine European countries. Front Neurol. (2018) 9:585. 10.3389/fneur.2018.00585 30087651 PMC6067090

[28] Dolejs J Homolkova H. Why does child mortality decrease with age? Modeling the age-associated decrease in mortality rate using WHO metadata from 14 European countries. Front Pediatrics. (2020) 8:2296–360. 10.3389/fped.2020.527811 PMC7653179 33194882

[29] Halley E. An estimate of the degrees of mortality of mankind, drawn from curious tables of the births and funerals at the city of Breslaw, with an attempt to ascertain the price of annuities on lives. Philos Trans. (1693) 17:596–610. 10.1098/rstl.1693.0007

[30] Bellhouse DR. A new look at Halley's life table. J R Statist Soc A. (2011) 174:823–32. 10.1111/j.1467-985X.2010.00684.x

[31] Luy MA Wittwer-Backofen U. The halley band for paleodemographic mortality analysis. In: Bocquet-Appel JP, editor. Recent Advances in Palaeodemography. Dordrecht: Springer (2008). p. 119–41. 10.1007/978-1-4020-6424-1_5

[32] World Health Organization. Mortality ICD10. (2018). Available online at: https://www.who.int/data/gho/data/themes/mortality-and-global-health-estimates/download-the-raw-data-files-of-the-who-mortality-database (accessed March 15, 2018).

[33] World Health Organization. The International Classification of Diseases, 10th Revision, 3 digit codes. (1997). Available online at: http://apps.who.int/classifications/apps/icd/icd10online (accessed January 21, 2017).

[34] Bureau of the Census (2019). Available online at: https://www.census.gov/data-tools/demo/idb/#/country (accessed May 15, 2019).

[35] Lu TH Lunetta P Walker S. Quality of cause-of-death reporting using ICD-10 drowning codes: a descriptive study of 69 countries. BMC Med Res Methodol. (2010) 10:1471–2288. 10.1186/1471-2288-10-30 PMC2858216 20374660

[36] Daking L Dodds L. ICD-10 mortality coding and the NCIS: a comparative study. Health Inf Manag. (2007) 36:11–23. 10.1177/183335830703600204 18195402

[37] Stolpe S Kowall B Stang A. Decline of coronary heart disease mortality is strongly effected by changing patterns of underlying causes of death: an analysis of mortality data from 27 countries of the WHO European region 2000 and (2013). Eur J Epidemiol. (2021) 36:57–68. 10.1007/s10654-020-00699-0 33247420 PMC7847455

[38] Loane M Dolk H Garne E Greenlees R. Paper 3: EUROCAT data quality indicators for population-based registries of congenital anomalies. Birth Defects Res A-Clin Mol Teratol. (2011) 91:23–30. 10.1002/bdra.20779 21384530

[39] Dolk H Loane M Garne E. The prevalence of congenital anomalies in Europe. In: Posada de la Paz M, Groft S, editors. Rare Diseases Epidemiology. Dordrecht: Springer Netherlands (2010). p. 349–64. 10.1007/978-90-481-9485-8_20 20824455

[40] Heude B Scherdel P Werner A Le Guern M Gelbert N Walther D. A big-data approach to producing descriptive anthropometric references: a feasibility and validation study of paediatric growth charts. Lancet Digital Health. (2019) 1:2589–7500. 10.1016/S2589-7500(19)30149-9 33323223

[41] Albright FS Orlando P Pavia AT Jackson GG Cannon Albright LA. Evidence for a heritable predisposition to death due to influenza. J Infect Dis. (2008) 197:18–24. 10.1086/524064 18171280

[42] Ward JL Wolfe I Viner RM. Cause-specific child and adolescent mortality in the UK and EU15+ countries. Arch Dis Child. (2019) 105:1055–60. 10.1136/archdischild-2019-318097 32847797

[43] Health Policy Team. Why Children Die - Research and Recommendations. Royall College of Paediatrics and Child Health. (2014). Available online at: https://www.rcpch.ac.uk/resources/why-children-die-research-recommendations (accessed September 10, 2020).

