# Supplementary material for: Why Does Child Mortality Decrease With Age? Modeling the Age-Associated Decrease in Mortality Rate Using WHO Metadata From 25 Countries
Source: Front Pediatr. 2021 Aug 11;9:657298. doi: 10.3389/fped.2021.657298 (PMC8387124; doi:10.3389/fped.2021.657298)
Supplement: Supplementary file 10 [file Data_Sheet_1.PDF]

Why does child mortality decrease with age? Modeling the age-associated decrease in mortality rate using WHO metadata from 25 countries

Appendix

Age trajectory of total mortality

Because the adjusted coefficients of determinations calculated for ATTM in the two parametric linear model for $\overline{R}^2$ were high in Japan and Peru (0.9948 in Japan and 0.9929 in Peru) the lower value of $\mathbf{R_b^2}$ was due to slopes which differ from the value -1. ATTM in Peru and Japan were also exceptional with respect to age category of the minimal mortality value (the minimal value of ATTM was reached in the age category [10, 15) years in the two populations). The explanation of the results is difficult without any speculation. One possibility is that the slower decrease was related to Japanese population which is known for high life expectancy. The effect was less significant in Peru (with steeper decrease than in Japan), and simultaneously, population in Peru contains subpopulation of Japanese (Japan Peruvians) which constituting approximately 1.4% of the population of Peru. The speculation may show that the slower mortality decrease may be exclusive to Japanese population.

Age trajectory of mortality due to CACNS

ATM due to CACNS differed from other diseases because they reached the minimal value in higher ages and the inverse proportion model was valid with high coefficients of determination.[26-28] The age range [0, 15) years was used here to compare ATM from CACNS as, in all populations studied, this was the lowest age range in which mortality decreased. All 32 ATM due to CACNS were available in the age range [0, 15) years. Linearity was rejected in **nine** countries (Germany, Italy, Austria, Poland, Slovakia, Finland, Australia, New Zealand and USA), while it was not rejected in all other countries and in all aggregated populations. In the next step, the inverse proportion was tested in the rest 16 countries and all 7 aggregated populations (in ATM due to CACNS where the linearity was confirmed). The inverse proportion was rejected only in France, Czech Republic, Hungary and Brazil. Consequently, the inverse proportion was confirmed in 12 countries and in all aggregated populations in the age range [0, 15) years. The coefficients of determination calculated in the model of inverse proportion $\mathbf{R_b^2}$ were very high, in general, reaching the maximum value of 0.9942 in the largest population P25, and the value of 0.9937 in P14. All results are shown in **Table 2** here. The coefficients of determination $\mathbf{R_b^2}$ calculated for ATM from CACNS were almost as high as those calculated for total mortality (Table 1 in main text). For example, the coefficient of determination was 0.9965, for total mortality within the age range [0, 10) years in P25, while a value of 0.9938 for ATM from CACNS was found in P25 in the same age range. ATM from CACNS decreased up 60 years in the largest population P25 and the coefficient of determination $\mathbf{R_b^2}$ reached 0.9953 the age range [0, 60) years in P25. The ATM is shown in Figure 2 and all other ATM due to CACNS are shown in file "**All_Populations_CACNS_Animation_2.mp4"** in supplementary material.

Table 2 Results calculated in CACNS during the first 15 years in all populations in the log-log scale.

Population	Test of linearity	γ	Lower CI 95%	Upper CI 95%	μ_1	$\overline{R}^2$	R_b^2	Test of c/x
France	↑ 0.75	-1.092	-1.180	-1.004	0.92	0.9891	0.9832	↓ 0.04
Germany	↓ 0.02							
Italy	↓ 0.03							
Spain	↑ 0.65	-0.937	-1.020	-0.854	1.33	0.9869	0.9839	↑ 0.12
UK	↑ 0.08	-1.051	-1.112	-0.989	1.68	0.9943	0.9926	↑ 0.09
**P1**	↑ 0.13	-0.998	-1.062	-0.934	1.41	0.9931	0.9938	↑ 0.95
Czech Republic	↑ 0.14	-0.883	-0.951	-0.815	2.49	0.9901	0.9739	↓ 0.00
Austria	↓ 0.02							
Hungary	↑ 0.37	-0.827	-0.911	-0.744	3.54	0.9830	0.9421	↓ 0.00
Poland	↓ 0.04							
Slovakia	↓ 0.03							
**P2**	↑ 0.12	-0.943	-1.013	-0.872	3.33	0.9907	0.9880	↑ 0.10
Sweden	↑ 0.34	-0.939	-1.028	-0.851	1.53	0.9852	0.9827	↑ 0.15
Norway	↑ 0.19	-0.954	-1.086	-0.822	1.64	0.9686	0.9699	↑ 0.44
Denmark	↑ 0.15	-0.991	-1.060	-0.923	2.14	0.9920	0.9928	↑ 0.78
Finland	↓ 0.01							
**P3**	↑ 0.08	-0.949	-1.040	-0.859	1.71	0.9848	0.9837	↑ 0.23
**P14**	↑ 0.07	-0.975	-1.035	-0.915	1.82	0.9937	0.9937	↑ 0.36
Argentina	↑ 0.43	-1.071	-1.159	-0.982	3.95	0.9886	0.9856	↑ 0.10
Brazil	↑ 0.73	-1.096	-1.160	-1.032	4.17	0.9942	0.9872	↓ 0.01
Peru	↑ 0.75	-1.042	-1.097	-0.987	2.46	0.9953	0.9942	↑ 0.11
Venezuela	↑ 0.23	-0.974	-1.043	-0.904	5.97	0.9915	0.9917	↑ 0.40
Chile	↑ 0.10	-1.068	-1.167	-0.969	4.50	0.9858	0.9833	↑ 0.15
Colombia	↑ 0.08	-1.080	-1.168	-0.993	3.04	0.9889	0.9846	↑ 0.07
**P4**	↑ 0.90	-1.005	-1.081	-0.929	3.84	0.9905	0.9915	↑ 0.88
Mexico	↑ 0.78	-1.034	-1.101	-0.967	5.04	0.9930	0.9927	↑ 0.27
Japan	↑ 0.27	-0.941	-1.042	-0.840	0.82	0.9810	0.9792	↑ 0.21
Australia	↓ 0.02							
New Zealand	↓ 0.02							
USA	↓ 0.01							
**P5**	↑ 0.16	-1.030	-1.093	-0.968	2.43	0.9938	0.9937	↑ 0.30
**P25**	↑ 0.46	-1.037	-1.094	-0.980	2.81	0.9949	**0.9942**	↑ 0.17

Notes: *The column labeled as "***Test of linearity***" contains p-values of the test of linearity. The column labelled as "slope γ" contains point estimation of the slope in the linear two parametric model in the log-log scale and the next two columns ("Lower" and "Upper") contain the limits of 95% confidence intervals of the parameter γ. The parameter μ_1 is per 100,000 living per one year and was calculated in the two parametrical linear model. $\overline{R}^2$ is the adjusted coefficient of determination calculated for one predictor and ten points in the linear two parametrical model. R_b^2 is the coefficient of determination calculated for the inverse proportion with a single parameter in the log-log scale (it was automatically also the adjusted coefficient of determination).*[28] *The last column labelled as "***c/x***" contains p-value of the standard Fisher's test, which determined that the linear model with two parameters does not provide a significantly better fit than the inverse proportion with a single parameter (which may slowly differ from the values of μ_1 calculated in the two parametrical linear model).*

Bending ATM

Diseases from chapters for which the decrease in mortality was slower during the first year than after the first year and were not related to congenital impairment, were aggregated to the group labeled as "Other diseases”. This group contained the chapters I-XV, without the chapter II (Neoplasms). ATM due to dominant chapters XVI, XVII with congenital impairment or impairment originating in the perinatal period decreased during the whole age interval [0, 15) years and were not bending. ATM due to symptoms, signs and abnormal clinical and laboratory findings, not elsewhere classified (Chapter XVIII) were studied separately for problematic determination of death cause. Besides, the last three chapters of external causes (XIX, XX, and XXI) were aggregated into single category labeled as "Accidents”.

Bending ATM from specific diseases represented the next empirical verification of TCIR. All following files may be found in supplementary material. ATM due to "Other diseases" in all 32 populations are shown in file "**All_Populations_Other_diseases_Animation_3.mp4**". ATM due to the first chapter "Certain infectious and parasitic diseases" of ICD10 in all populations are shown in file "**All_Populations_Chapter_1_Animation_4.mp4**". ATM due to all chapters in two populations P14 and P25 are shown in file "**All_Chapters_P25_and_P14_Animation_5.mp4**".

At first, it was difficult to assume that any hypothetical individual development was more significant after the age of 1 or 2 years than during the first year (it had to be valid if any homogenous description of population was assumed to explain the bending ATM). Such hypothetical homogenous development had to be significantly slowed down during the first year and more significant after the first year, and such explanation was sidelined here. It was also shown that the spectrum of diseases strongly changed during the first ten years. [26, 28] For example, 94% deaths were registered in two dominant chapters "Certain conditions originating in the perinatal period" (XVI) and "Congenital malformations, deformations and chromosomal abnormalities" (XVII) in the first 28 days in aggregated population P25 (in the first three points of ATM) while it was only 7% in the age interval [5, 10) years. Similar results were detected in all other 31 populations (data of all populations aggregated in specific calendar period may be found in the file "**25_Countries_Dolejs_Homolkova.xlsx**"). Figure 4 illustrates the proportions of deaths from diseases categorized in specific chapters of ICD10 in all age categories.

Figure 4 Proportions of deaths from diseases categorized in specific chapters of ICD10 in the largest population P25.

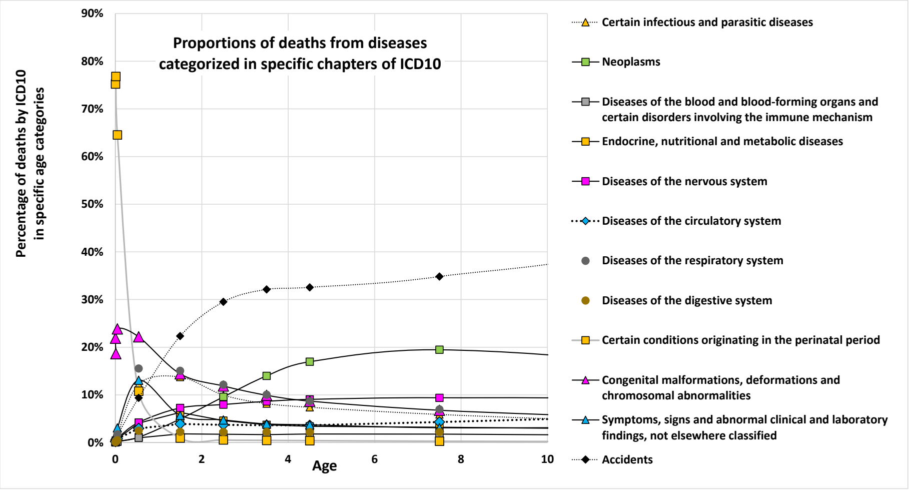

In contrast, the TCIR explained the child mortality decrease with age based on the sequential extinction of more severe impairments. It enabled also the explanation of the bending ATM and formula (2) was derived in the previous study according to TCIR.[26-28]

$$\mu(x) = \frac{\mu_1}{x}.\left[1 - e^{(-r_{max}.x)}\right], and\ for\ small\ x: \cong \frac{\mu_1}{x}\left[1 - (1 - r_{max}.x)\right] = \mu_1.r_{max} \quad (2)$$

If the maximal congenital individual risk of death r_{max} in born population was not big then the element in the squared brackets differ from the value 1 for small x.

For example, only less sever impairments may be registered in the fifth chapter of ICD10 "Diseases of the nervous system" because more sever impairments may be registered in CACNS, and consequently r_{max} might be small in the fifth chapter "Diseases of the nervous system". On the other hand, if r_{max} is big then the element in squared brackets equals to one (the exponential element is very small), and consequently, formula (2) crosses to the model of the inverse proportion with single parameter (it was discussed in more details in previous studies). [25-26, 28] If r_{max} is small and simultaneously age is small then the element in squared brackets equals to product $r_{max}.x$ and the whole formula equals to product $\mu_1.r_{max}$ and ATM is age-independent for small x.

ATM due to more chapters decreased according to the inverse proportion after the first year in the age interval [1, 10) years. In the largest population P25, ATM were constructed in 16 of the first 18 chapters (two chapters were not relevant to the study because zero death were registered in some age categories even in the largest population P25). The inverse proportion was not rejected in 8 from 16 ATM in P25 in the age interval [1, 10) years. Age-independence was not rejected in 6 from 16 ATM in P25 in the age interval [0, 365) days. It was confirmed that ATM decreased slowly during the first year and decreased steeply after the first year. The results are presented here in **Table 3**.

Besides, nonlinear method of least squares was used to fit data using the formula (2), and parameters μ_1 and r_{max} were calculated. The results are also shown in **Table 3** (**Table 3** contains also the number of deaths in P25 in specific chapter of the ICD10 within the age interval [0, 10) years and its proportions to all deaths in the age range). Adjusted coefficient of determinations calculated for formula (2) were lower than for ATTM and ATM due to CACNS calculated in the inverse proportion in all populations. Relatively higher values calculated using formula (2) were detected in "Diseases of the genitourinary system (XIV.)" (with $\bar{R}^2$=0.98), "Diseases of the skin and subcutaneous tissue (XII.)" (with $\bar{R}^2$=0.97) and in the aggregated group "Other diseases" (with $\bar{R}^2$=0.94). How the formula (2) was successful is also shown visually in presentations in the files:
"**All_Populations_Other_diseases_Animation_3.mp**",
"**All_Populations_Chapter_1_Animation_4.mp4**",
"**All_Chapters_P25_and_P14_Animation_5.mp4**".

Table 3. Results calculated in main chapters of ICD10 during the first 10 years in P25.

Chapter	ΣDi	P	ATM	the 1st day-365 days γ_1	γ_1=0	γ_1=-1	1-10 years γ_2	γ_2=0	γ_2=-1	r_{max}	μ_1	$\bar{R}^2$
Certain infectious and parasitic diseases (I.)	129,937	5.72	yes	-0.20	↑0.14	↓0.01	-1.40	↓0.00	↓0.05	6.7	10.1	0.91
Neoplasms (II.)	78,772	3.47	yes	-0.60	↓0.04	↑0.08	-0.01	↑0.93	↓0.00	x	x	x
Diseases of the blood and blood-forming organs and certain disorders involving the immune mechanism (III.)	16,484	0.73	yes	-0.39	↓0.05	↓0.02	-0.89	↓0.00	↑0.38	6.7	1.8	0.88
Endocrine, nutritional and metabolic diseases (IV.)	52,602	2.31	yes	-0.33	↓0.05	↓0.01	-1.31	↓0.00	↑0.17	10.8	4.5	0.94
Mental and behavioural disorders (V.)	841	0.04	yes	-0.81	↓0.04	↑0.38	-0.34	↑0.05	↓0.01	7.3	0.1	0.37
Diseases of the nervous system (VI.)	74,707	3.29	yes	-0.25	↓0.01	→0.00	-0.71	↓0.00	↓0.03	**3.9**	8.6	0.94
Diseases of the eye and adnexa (VII.)	10	0.00	no	x	x	x	x	x	x	x	x	x
Diseases of the ear and mastoid process (VIII.)	759	0.03	yes	-0.50	↑0.13	↑0.12	-1.05	↓0.01	↑0.80	11.5	0.1	0.79
Diseases of the circulatory system (IX.)	44,621	1.96	yes	-0.50	↓0.02	↓0.02	-0.83	↓0.01	↑0.34	20.5	3.8	0.92
Diseases of the respiratory system (X.)	157,564	6.93	yes	-0.07	↑0.28	→0.00	-1.36	↓0.00	↓0.03	4.8	12.0	0.92
Diseases of the digestive system (XI.)	29,306	1.29	yes	-0.37	↑0.06	↓0.02	-0.89	↓0.00	↑0.33	11.7	2.5	0.91
Diseases of the skin and subcutaneous tissue (XII.)	1,163	0.05	yes	-0.24	↓0.01	→0.00	-0.86	↓0.01	↑0.45	5.8	0.1	0.97
Diseases of the musculoskeletal system and connective tissue (XIII.)	1,851	0.08	yes	-0.50	↑0.06	↑0.06	-0.20	↑0.20	↓0.01	5.5	0.2	0.66
Diseases of the genitourinary system (XIV.)	11,044	0.49	yes	-0.39	↑0.05	↓0.02	-0.73	↓0.01	↑0.08	17.1	1.0	0.98
Pregnancy, childbirth and the puerperium (XV.)	0	0.00	no	x	x	x	x	x	x	x	x	x
Certain conditions originating in the perinatal period (XVI.)	896,504	39.44	yes	-1.25	↓0.01	↑0.19	-1.61	↓0.00	↓0.03	x	x	x
Congenital malformations, deformations and chromosomal abnormalities (XVII.)	428,862	18.87	yes	-0.92	↓0.00	↓0.04	-1.35	↓0.00	↓0.02	x	x	x
Symptoms, signs and abnormal clinical and laboratory findings, not elsewhere classified (XVIII.)	121,605	5.35	yes	-0.52	↓0.04	↓0.05	-1.26	↓0.00	↑0.10	140.8	4.9	0.92
Accidents (XIX,.XX.,XXI.)	226,437	9.96	yes	-0.45	↑0.10	↑0.07	-0.61	↓0.00	↓0.00	x	x	x
"Other diseases"	520,889	22.92	yes	-0.27	↑0.05	→0.01	-1.11	↓0.00	↑0.45	7.5	45.8	0.94
CACNS (ICD10 codes: Q00-Q07)	63,067	2.77	yes	-1.00	↓0.01	↑1.00	-1.03	↓0.00	↑0.66	x	x	x

Notes: *Diseases from chapters for which the decrease in mortality was slower during the first year than after the first year and were not related to congenital impairment were added to the group labeled as "Other diseases." This group contained the chapters I-XV, without the chapter II (neoplasms). The last three chapters of ICD10 (XIX, XX, and XXI) were aggregated into a category labeled as "Accidents." The column labeled as "**ΣDi**" contains number of deaths within the age range [0, 10) years. The column labeled as "**P**" contains proportions of diseases of specific chapter or chapters in all deaths within the age interval [0, 10) years. The column labeled as "**ATM**" indicates if all age categories contained a non-zero number of deaths and if ATM could be constructed in P25 for the age range [0, 10) years. If all age categories contained a non-zero number of deaths, the row was marked with "yes" in the column "**ATM**". If one age category contained zero deaths, the row was marked with "no" in the column "**ATM**." Slope* γ_1 *was calculated in the log-log scale for the first four age categories (during the first year). Slope* γ_2 *was calculated in the log-log scale for the age interval [1, 10) years. The column labeled as "*$\gamma_1=0$*" contains p-value of the test of the null hypothesis Ho:* $\gamma_1=0$*. The column labeled as "*$\gamma_1=-1$*" contains p-value of the test of the null hypothesis Ho:* $\gamma_1=-1$*. The column labeled as "*$\gamma_2=0$*" contains p-value of the test of the null hypothesis Ho:* $\gamma_2=0$*. The column labeled as "*$\gamma_2=-1$*" contains p-value of the test of the null hypothesis Ho:* $\gamma_2=-1$*. The columns labeled as "*r_{max}*" and "*μ_1*" contain point estimations of the two parameters calculated using model (2).* $\bar{R}^2$ *is adjusted coefficient of determination calculated for two parameters and nine points in the model (2).*

ATM due to neoplasms

It was described in previous studies that ATM due to neoplasms significantly differed from other ATM.[25-26, 28] Similar ATM were detected only in two insignificant chapters "Mental and behavioural disorders (V.)" and "Diseases of the musculoskeletal system and connective tissue (XIII.)". The two chapters were insignificant because their proportions in all deaths in the age interval [0, 10) years were less than 0.04 % in P25 (similar proportions were in other 31 populations; the minimal proportion of chapter V. was zero in Slovakia and the maximal proportion was 0.21% in Hungary; the minimal proportion of chapter XIII. was 0.02% in the Czech Republic and the maximal proportion was 0.29% in New Zealand).

The proportion of neoplasms was about 3.5% in P25 while the minimal proportion of neoplasms in all deaths in countries in the age interval [0, 10) years was 2.5% in Venezuela and the maximal value was 7.7% in Sweden. More detail data are in file "**25_Countries_Dolejs_Homolkova.xlsx**". Resulting ATM from neoplasms of all populations are shown in file "**All_Populations_Neoplasms_Animation_6.mp4**".

Shapes of these ATM were practically identical in all populations in all continents. They decreased during the first month of life and became age-independent in the age interval [28 days, 15 years). Two basic shapes in the log-log scale: "age-independence" and "inverse proportion" were tested in all populations in the age interval [0, 28 days) (in the first three age categories). Namely, null hypothesis Ho: slope=-1 and Ho: slope=0 were evaluated. The same null hypothesis in the log-log scale in all populations were tested in the age interval [28 days, 15 years). The results are shown in Table 4 here. Slope -1 was rejected in the interval [0, 28 days) only in P1, Argentina and Venezuela where the decrease was slower. The inverse proportion model was not rejected in all other 29 populations in the interval [0, 28 days). Simultaneously, age-independence was rejected in the age interval [28 days, 15 years) only in Italy (with positive slope +0.12) and in Slovakia (with negative slope -0.2), and was not rejected in all other 30 populations. Consequently, it was detected that ATM from neoplasms were age-independent in the age range [28 days, 15 years) (the two exceptions had opposite sign of slope). More detail results are shown in Table 4.

Table 4 Results calculated in neoplasms in all populations.

Population	Neoplasms per all deaths	Neoplasms per 10^5 living	c/x during the first month	$\overline{R}_1^{\,2}$	Age-independence during (28 days, 15 years]	1-CV
France	0.0572	2.91	↑ **0.59**	0.9811	↑ **0.57**	0.990
Germany	0.0550	2.56	↑ **0.38**	0.9926	↑ **0.91**	0.993
Italy	0.0715	3.24	↑ **0.55**	0.9565	↓ **0.04**	0.984
Spain	0.0691	3.43	↑ **0.17**	0.8913	↑ **0.19**	0.991
UK	0.0452	2.74	↑ **0.07**	0.9963	↑ **0.52**	0.992
**P1**	**0.0569**	**2.90**	↓ **0.03**	**0.9998**	↑ **0.25**	**0.993**
Czech Republic	0.0649	3.55	↑ **0.08**	0.9967	↑ **0.45**	0.987
Austria	0.0484	2.30	↑ **0.68**	0.8241	↑ **0.71**	0.983
Hungary	0.0531	4.36	↑ **0.74**	0.6919	↑ **0.05**	0.986
Poland	0.0475	3.57	↑ **0.13**	0.9977	↑ **0.58**	0.987
Slovakia	0.0430	2.73	↑ **0.30**	0.6586	↓ **0.03**	0.975
**P2**	**0.0506**	**3.50**	↑ **0.27**	**0.9833**	↑ **0.17**	**0.990**
Sweden	0.0769	2.98	↑ **0.87**	0.9446	↑ **0.80**	0.982
Norway	0.0621	2.76	↑ **0.41**	0.9941	↑ **0.36**	0.978
Denmark	0.0607	2.64	↑ **0.86**	0.8821	↑ **0.15**	0.987
Finland	0.0751	3.09	↑ **0.27**	0.9829	↑ **0.53**	0.991
**P3**	**0.0697**	**2.89**	↑ **0.56**	**0.9934**	↑ **0.76**	**0.988**
**P14**	**0.0561**	**3.02**	↑ **0.09**	0.9989	↑ **0.65**	**0.993**
Argentina	0.0258	4.11	↓ **0.05**	0.9970	↑ **0.70**	0.994
Brazil	0.0263	4.25	↑ **0.21**	0.9394	↑ **0.15**	0.992
Peru	0.0265	3.95	↑ **0.26**	0.9089	↑ **0.30**	0.987
Venezuela	0.0250	5.39	↓ **0.02**	0.9997	↑ **0.75**	0.989
Chile	0.0387	4.04	↑ **0.16**	0.9871	↑ **0.24**	0.988
Colombia	0.0321	5.15	↑ **0.52**	0.9330	↑ **0.60**	0.991
**P4**	**0.0270**	**4.42**	↑ **0.10**	**0.9900**	↑ **0.60**	**0.993**
Mexico	0.0296	5.51	↑ **0.21**	0.9934	↑ **0.97**	0.994
Japan	0.0614	2.47	↑ **0.43**	0.9176	↑ **0.72**	0.995
Australia	0.0517	2.95	↑ **0.09**	0.9996	↑ **0.66**	0.987
New Zealand	0.0417	3.09	↑ **0.64**	0.9837	↑ **0.48**	0.983
USA	0.0320	2.84	↑ **0.51**	0.9795	↑ **0.53**	0.993
**P5**	**0.0354**	**3.31**	↑ **0.42**	0.9790	↑ **0.99**	**0.994**
**P25**	**0.0347**	**3.56**	↑ **0.21**	**0.9895**	↑ **0.91**	**0.994**

Notes: *The first column contains proportion of deaths due to neoplasms in the age interval [0, 10) years per one death in the age interval (the age interval was used because it was used in all other chapters in Table 3). The second column contains proportions of deaths due to neoplasms per 100,000 living in in the age interval [0, 10) years. The column labeled as "**c/x during the first month**" contains p-values of the test of the null hypothesis Ho: γ= -1 in the log-log scale in the age interval [0, 28) days. $\overline{R}_1^2$ is the adjusted coefficient of determination calculated for one predictor and four points in the linear two parametrical model in the age interval [0, 28) days. The column labeled as "Age-independence during [28 days, 15 years) contains p-values of the test of the null hypothesis Ho: γ=0 in the log-log scale in the age interval [28 days, 15 years). The last column labeled as "1-CV" contains values calculated as "one minus coefficient of variation (CV)". CV was calculated as the ratio of the standard deviation and the mean of logarithm of mortality rates in the age interval [28 days, 15 years). The values in the last column may be used to evaluated residuals in the model of age-independence.*

It has to be stressed that ATM due to neoplasms were constructed for the whole second chapter of ICD10. It was previously shown that two subcategories "Malignant neoplasms, stated or presumed to be primary, of specified sites, except of lymphoid, haematopoietic and related tissue (C00-C75)" and "Malignant neoplasm of brain (C71)" in the age interval [0, 15) years were dominant in the second chapter.[26] More detailed study may show if the same results are valid in specific subcategories. For example, it may be successful in the largest population P25 using the Halley method in more detail subcategories.

The evidence that mortality due to neoplasms decreased with age in the first month and was age-independent within the age range [28 days, 15 years) may be important for the verification of any hypothesis that a neoplasm is significant only to a subpopulation with some congenital predisposition (e.g. a genetically susceptible subpopulation). According to TCIR, such subpopulation should be larger than the sum of all deaths in the age interval [28 days, 15 years), since no extinction was observed (no decrease in ATM). The sum of all deaths within the age range [28 days, 15 years) simply represents the lower limit of the subpopulation assumed to be susceptible to the disease. The interpretation is based on all ATM results and it is very easy to calculate the limit. On the other hand, such number is only the lower limit of the hypothetical subpopulation. This means that if any predisposition exists then considered disease has to relate subpopulation large than the sum of all death in the whole specific population. For example, the proportion of all deaths due to conditions of the chapter "Neoplasms" within all living people in P25 was about 3.56 per 100,000, which means that the incidence of these conditions in a hypothetical subpopulation should be higher (the minimal proportion was 2.30 in Austria and the maximal was 5.51 in Mexico).

ATM with fast decrease in the first year

The two dominant chapters "Certain conditions originating in the perinatal period" (XVI) and "Congenital malformations, deformations and chromosomal abnormalities" (XVII) addressed cases of congenital abnormality or any other defect developed during the perinatal period. Diseases of the two chapters accounted for 39% and 23% of all deaths in P25 within the age interval [0, 10) years and ATM constructed in the two chapters strongly decreased in the age interval. These ATM in all populations are shown in two animations in two files: "**All_Populations_Certain_conditions_in_perinatal_period_Animation_7.mp4" and "All_Populations_CA_Animation_8.mp4**".

ATM due to chapter "Certain conditions originating in the perinatal period" (XVI.)

ATM due to chapter "Certain conditions originating in the perinatal period" decreased steeply than other ATM. It was previously shown that slope was near -2 in the log-log scale. It was tested here in two age intervals [0, 15) years and [1, 5) years and the model c/(x.x) which has slope -2 in the log-log scale was confirmed. For example, the slope -2 was not rejected in the age interval [1, 5) years and was rejected in the age interval [0, 15) years in P25 (p-value was near the significance level 0.05 for the wider age interval [0, 15) years). The results calculated in all population in two age intervals are shown in Table 5. The linear model with slope -2 in the log-log scale was rejected only in three ATM of 23 ATM in the age interval [1, 5) years (only 23 ATM were constructed for the chapter XVI.). Two of the three exceptions were due to slower mortality decrease (Denmark and USA) and ATM in Chile was steeper. For example, the linearity was not rejected in the age interval [1 day, 30 years) in population P25 and point estimation of slope was 1.80 with a 95% CI of (-2.03, -1.57), and with the adjusted coefficient of determination 0.965.

Meaning of the specific slope -2 was theoretically explained in TCIR and the results detected in 25 countries and the results detected in aggregated populations confirmed TCIR. If congenital individual risks of death are approximately equally frequent or if they are distributed according to the normal distribution with high variation then theoretical ATM according to TCIR decreases with the slope -2 in the log-log scale. The explanation may be expressed: "Impairments originating in the perinatal period were not affected by any previous selection (during the prenatal period or during the previous generations), and consequently, more sever impairments may be equally frequent as less sever impairments".[25-26, 28] Such mechanisms may be valid after huge accident if more sever impairments are not suppressed and are not less probable than less sever impairments.

Table 5 Results calculated in the chapter "Certain conditions originating in the perinatal period" (XVI.) calculated in all populations the log-log scale.

Population	Age interval [0, 15) years						Age interval [1, 5) years					
	Test of linearity	γ	Lower CI 95%	Upper CI 95%	$\bar{R}^2$	Test of c/(x,x)	Test of linearity	γ	Lower CI 95%	Upper CI 95%	$\bar{R}^2$	Test of c/(x,x)
France	↑ 0.11	-1.73	-2.03	-1.42	0.950	↑0.07	↑ 0.16	-2.39	-4.95	0.17	0.780	↑0.58
Germany	↑ 0.20	-1.58	-1.79	-1.36	0.970	↓0.00	↑ 0.57	-1.60	-3.78	0.58	0.666	↑0.51
Italy	↑ 0.18	-1.59	-1.81	-1.36	0.968	↓0.00	↑ 0.20	-1.33	-3.75	1.08	0.479	↑0.36
Spain	↑ 0.21	-1.56	-1.81	-1.31	0.958	↓0.00	↑ 0.55	-1.84	-4.73	1.05	0.580	↑0.84
UK	↓ 0.03						↑ 0.93	-1.87	-2.33	-1.42	0.987	↑0.35
**P1**	↑ 0.12	-1.62	-1.84	-1.39	0.968	↓0.00	↑ 0.13	-1.73	-3.08	-0.39	0.878	↑0.48
Czech Republic												
Austria	↑ 0.71	-1.50	-1.72	-1.29	0.966	↓0.00	↑ 0.11	-1.55	-4.44	1.33	0.458	↑0.57
Hungary												
Poland	↓ 0.01						↑ 0.90	-3.05	-5.37	-0.74	0.884	↑0.19
Slovakia												
**P2**	↑ 0.07	-1.80	-2.13	-1.47	0.946	↑0.20	↑ 0.48	-2.46	-4.11	-0.81	0.907	↑0.35
Sweden	↑ 0.22	-1.42	-1.58	-1.26	0.979	↓0.00	↑ 0.97	-1.66	-4.55	1.24	0.504	↑0.66
Norway	↑ 0.17	-1.47	-1.64	-1.30	0.977	↓0.00	↑ 0.58	-1.45	-4.27	1.36	0.423	↑0.49
Denmark	↑ 0.53	-1.46	-1.60	-1.32	0.984	↓0.00	↑ 0.10	-0.34	-1.54	0.87	-0.158	↓0.03
Finland												
**P3**	↑ 0.15	-1.47	-1.61	-1.33	0.985	↓0.00	↑ 0.94	-1.34	-2.32	-0.37	0.893	↑0.10
**P14**	↑ 0.10	-1.63	-1.86	-1.40	0.966	↓0.01	↑ 0.17	-1.74	-2.89	-0.59	0.910	↑0.44
Argentina												
Brazil	↑ 0.07	-1.80	-2.09	-1.51	0.958	↑0.15	↑ 0.51	-2.00	-3.42	-0.58	0.897	↑1.00
Peru												
Venezuela												
Chile	↓ 0.02						↑ 0.82	-2.37	-2.68	-2.06	0.996	↓0.04
Colombia	↑ 0.14	-1.73	-2.01	-1.44	0.956	↑0.06	↑ 0.36	-1.71	-2.97	-0.45	0.889	↑0.43
**P4**	↑ 0.07	-1.85	-2.15	-1.54	0.956	↑0.28	↑ 0.51	-2.04	-2.90	-1.17	0.962	↑0.87
Mexico												
Japan	↓ 0.00						↑ 0.38	-1.87	-2.73	-1.01	0.956	↑0.58
Australia	↑ 0.09	-1.63	-1.78	-1.48	0.986	↓0.00	↑ 0.79	-1.51	-2.57	-0.44	0.898	↑0.18
New Zealand												
USA	↑ 0.25	-1.64	-1.79	-1.49	0.987	↓0.00	↑ 0.14	-1.78	-1.95	-1.62	0.998	↓0.03
**P5**	↑ 0.05	-1.72	-1.92	-1.52	0.978	↓0.01	↑ 0.35	-1.79	-2.23	-1.36	0.987	↑0.18
**P25**	↑ 0.07	-1.75	-1.99	-1.50	0.967	↓0.05	↑ 0.14	-1.84	-2.52	-1.16	0.971	↑0.41

Notes: *The columns labeled as "**Test of linearity**" contains p-values of the test of the null hypothesis for the quadratic element H_o: $\delta = 0$ in quadratic model in the log-log scale. If the quadratic element was significant then the linear model was not calculated. If row is empty then ATM was not constructed in such population. The columns labeled as "γ" contains point estimations of the slopes in two parametrical linear model in the log-log scale. The next columns contain limits of 95% confidence interval of the parameter γ. $\bar{R}^2$ is adjusted coefficient of determination calculated for two parametrical linear models. The columns labeled as "**Test of c/(x.x)**" contains p-values of the standard Fisher's test that the two parametrical linear does not provide a significantly better fit than the same model with the slope -2 in the log-log scale (the model: c/(xx)).*

ATM due to "Congenital malformations, deformations and chromosomal abnormalities" (XVII)

Results calculated in two parametrical linear model in the chapter "Congenital malformations, deformations and chromosomal abnormalities" (XVII) in the age interval [0, 15) years) are in Table 6. ATM due to the chapter were constructed in all populations and the linearity was rejected in 8 populations (the Czech Republic, Slovakia, Denmark, Brazil, Venezuela, Colombia, P4 and Japan). The model of inverse proportion was tested in the rest 24 populations and was not rejected in 11 populations (Germany, Italy, Spain, P1, Austria, Hungary, Sweden, Norway, P14, Mexico, New Zealand). The mortality decrease was a little bit steeper in all ATM where the inverse proportion was (see the fifth column "Upper CI 95%" in Table 6). Slightly steeper ATM than the inverse proportion in the chapter may be more general result because arithmetic mean of slopes calculated in 25 countries (without aggregated populations) was -1.1 with the standard deviation 0.04 and with 95% C.I. (-1.11,-1.08). Similar result was detected in the largest population P25 where slope was γ = -1.12 with 95% C.I. (-1.21,-1.02) (see the last row in Table 6).

The results may be interpreted as a manifestation of small part of congenital impairments which were less selected before the birth according to TCRI. In other words, more sever impairments were more frequent if compared with the rule: “as more sever the impairment was than less frequent it was in born population”.

Table 6 Results calculated in the chapter "Congenital malformations, deformations and chromosomal abnormalities" (XVII.) in the age interval [0, 15) years in the log-log scale.

Population	Test of linearity	γ	Lower CI 95%	Upper CI 95%	$\overline{R}^2$	R_b^2	Test of c/x
France	↑ 0.17	-1.113	-1.217	-1.009	0.985	0.977	↓ 0.04
Germany	↑ 0.21	-1.069	-1.151	-0.986	0.990	0.987	↑ 0.09
Italy	↑ 0.11	-1.080	-1.185	-0.975	0.984	0.981	↑ 0.12
Spain	↑ 0.08	-1.049	-1.170	-0.927	0.978	0.978	↑ 0.38
UK	↑ 0.39	-1.123	-1.208	-1.038	0.990	0.980	↓ 0.01
**P1**	↑ 0.18	-1.090	-1.184	-0.997	0.988	0.982	↑ 0.06
Czech Republic	↓ 0.03						
Austria	↑ 0.95	-1.047	-1.136	-0.958	0.988	0.987	↑ 0.26
Hungary	↑ 0.07	-1.026	-1.127	-0.925	0.984	0.985	↑ 0.57
Poland	↑ 0.10	-1.115	-1.202	-1.027	0.990	0.980	↓ 0.02
Slovakia	↓ 0.01						
**P2**	↑ 0.10	-1.087	-1.173	-1.000	0.989	0.984	↓ 0.05
Sweden	↑ 0.48	-1.092	-1.211	-0.973	0.980	0.975	↑ 0.11
Norway	↑ 0.18	-1.063	-1.138	-0.988	0.992	0.989	↑ 0.09
Denmark	↓ 0.05						
Finland	↑ 0.95	-1.080	-1.151	-1.009	0.993	0.988	↓ 0.03
**P3**	↑ 0.32	-1.076	-1.152	-1.000	0.992	0.988	↓ 0.05
**P14**	↑ 0.16	-1.088	-1.177	-0.998	0.989	0.984	↑ 0.05
Argentina	↑ 0.06	-1.162	-1.271	-1.054	0.985	0.968	↓ 0.01
Brazil	↓ 0.04						
Peru	↑ 0.06	-1.099	-1.196	-1.002	0.987	0.980	↓ 0.05
Venezuela	↓ 0.03						
Chile	↑ 0.25	-1.159	-1.250	-1.068	0.990	0.972	↓ 0.00
Colombia	↓ 0.02						
**P4**	↓ 0.05						
Mexico	↑ 0.06	-1.101	-1.203	-0.999	0.986	0.979	↑ 0.05
Japan	↓ 0.03						
Australia	↑ 0.94	-1.156	-1.234	-1.079	0.992	0.975	↓ 0.00
New Zealand	↑ 0.87	-1.075	-1.162	-0.987	0.989	0.985	↑ 0.09
USA	↑ 0.60	-1.134	-1.220	-1.049	0.991	0.978	↓ 0.01
**P5**	↑ 0.14	-1.111	-1.201	-1.022	0.989	0.980	↓ 0.02
**P25**	↑ 0.08	-1.115	-1.211	-1.019	0.988	0.978	↓ 0.02

Notes: *The column labeled as "**Test of linearity**" contains p-values of the test of linearity. The column labelled as "slope γ" contains point estimation of the slope in the linear two parametric model in the log-log scale and the next two columns ("Lower" and "Upper") contain the limits of 95% confidence intervals of the parameter γ. $\overline{R}^2$ is the adjusted coefficient of determination calculated for one predictor and ten points in the linear two parametrical model. R_b^2 is the coefficient of determination calculated for the inverse proportion with a single parameter in the log-log scale (it was automatically also the adjusted coefficient of determination).[28] The last column labelled as "**c/x**" contains p-value of the standard Fisher's test, which determined that the linear model with two parameters does not provide a significantly better fit than the inverse proportion with a single parameter.*

ATM due to symptoms, signs and abnormal clinical and laboratory findings, not elsewhere classified (Chapter XVIII)

The chapter "Symptoms, signs and abnormal clinical and laboratory findings, not elsewhere classified" (XVIII) addressed cases with unclear diagnosis and was evaluated separately (it may be assumed that visible congenital abnormalities were not related to death in this chapter). A total of 5.94 % of all deaths due to these conditions in P25 occurred within the age range [0, 10) years.

A bending ATM was also detected in the chapter XVIII and ATM in P25 slowly decreased with slope -0.52 in the first year of life and decreased within the age range [1, 10), according to the inverse proportion (the null hypothesis slope = -1 was not rejected in P25 in the age interval [1, 10) years with P>0.10). The chapter may be formally also aggregated to group "Other diseases" but it was not jointed to the group because it contained cases with less clear diagnosis. ATM due to the chapter in two populations are shown in the file: "**All_Chapters_P25_and_P14_Animation_5.mp4**".

Age category "28-365 days"

Mortality rates in the age category "28-365 days" were systematically higher than the model of inverse proportion (see Figure 1 and animation in the file "**All_Populations_All_causes_Animation_1.mp4**"). It may be explained by the fact that arithmetic mean of limits of age category was used as one representative point of age category. If the numbers of deaths strongly decreased during the age category then arithmetic mean of limits of age category may be higher than actual expectation value of ages. The effect may be evaluated using the difference between the arithmetic mean and the logarithmic mean of limits of age category (if number of deaths decreases according to the inverse proportion then logarithmic mean is expectation value of ages).[26] These differences calculated in the age categories used in the WHO database are shown in Table 7. The methodical effect was more significant in the fourth age category.

Table 7 Differences between arithmetic and logarithmic mean.

Age category	Ai	Bi	Arithmetic mean	Logarithmic mean	Δ	Δ [%]
Infant deaths at age 0 day	0	0.003	0.001	x	x	x
Infant deaths at age 1-6 days	0.003	0.019	0.011	0.008	0.00	23
Infant deaths at age 7-27 days	0.019	0.077	0.048	0.042	0.01	13
Infant deaths at age 28-364 days	0.077	1	0.538	**0.360**	0.18	**33**
Deaths at age 1 year	1	2	1.5	1.443	0.06	4
Deaths at age 2 years	2	3	2.5	2.466	0.03	1
Deaths at age 3 years	3	4	3.5	3.476	0.02	1
Deaths at age 4 years	4	5	4.5	4.481	0.02	0
Deaths at age 5-9 years	5	10	7.5	7.213	0.29	4

Notes: Ai and Bi are limits of age category in years**.** Δ is absolute difference between arithmetic mean and logarithmic mean; Δ [%] is the difference between arithmetic mean and logarithmic mean divided by arithmetic mean in per cent.
