# Supplementary figures and images for: Why Does Child Mortality Decrease With Age? Modeling the Age-Associated Decrease in Mortality Rate Using WHO Metadata From 25 Countries
Source: Front Pediatr. 2021 Aug 11;9:657298. doi: 10.3389/fped.2021.657298 (PMC8387124; doi:10.3389/fped.2021.657298)

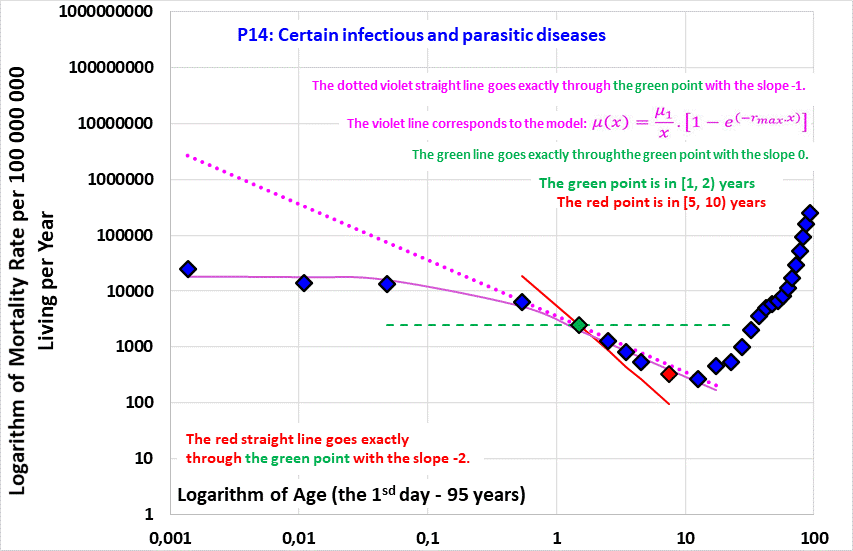

Supplement: Supplementary file 11 [file Data_Sheet_2.zip › All_Chapters_P25_and_P14_Animation_5.gif]

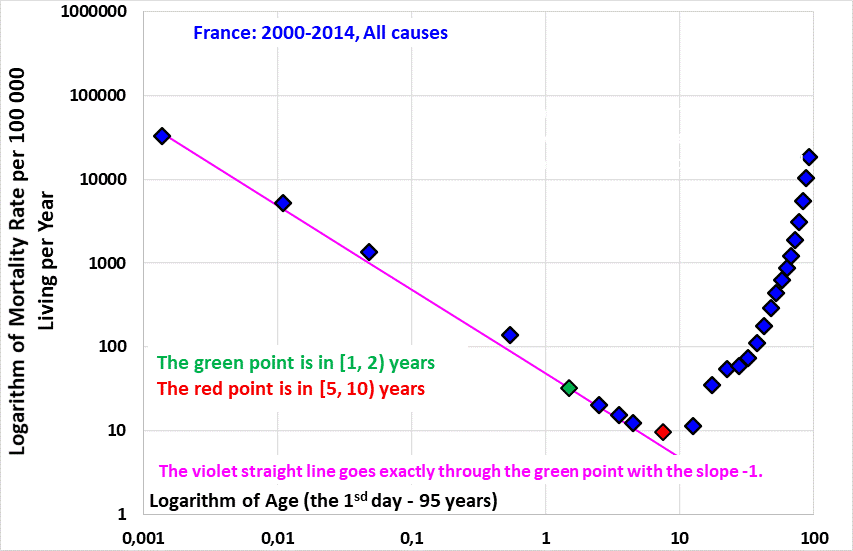

Supplement: Supplementary file 11 [file Data_Sheet_2.zip › All_Populations_All_causes_Animation_1.gif]

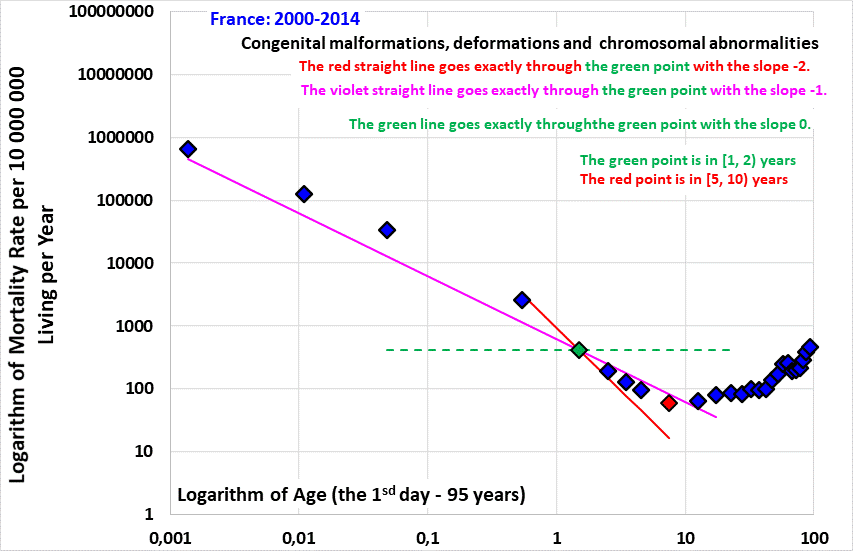

Supplement: Supplementary file 11 [file Data_Sheet_2.zip › All_Populations_CA_Animation_8.gif]

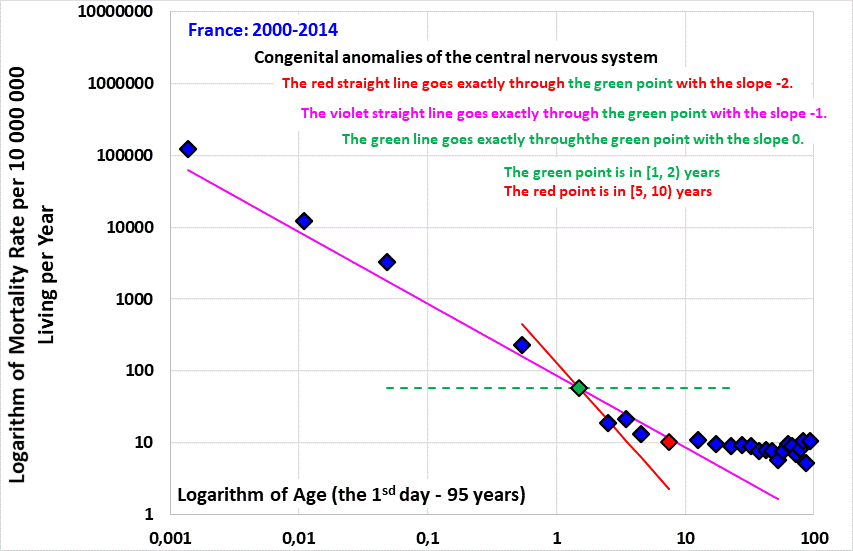

Supplement: Supplementary file 11 [file Data_Sheet_2.zip › All_Populations_CACNS_Animation_2.gif]

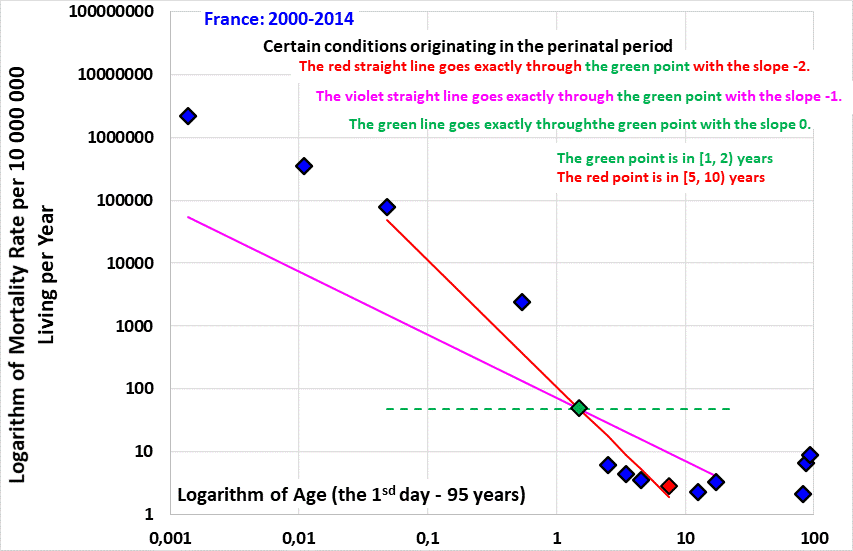

Supplement: Supplementary file 11 [file Data_Sheet_2.zip › All_Populations_Certain_conditions_in_perinatal_period_Animation_7.gif]

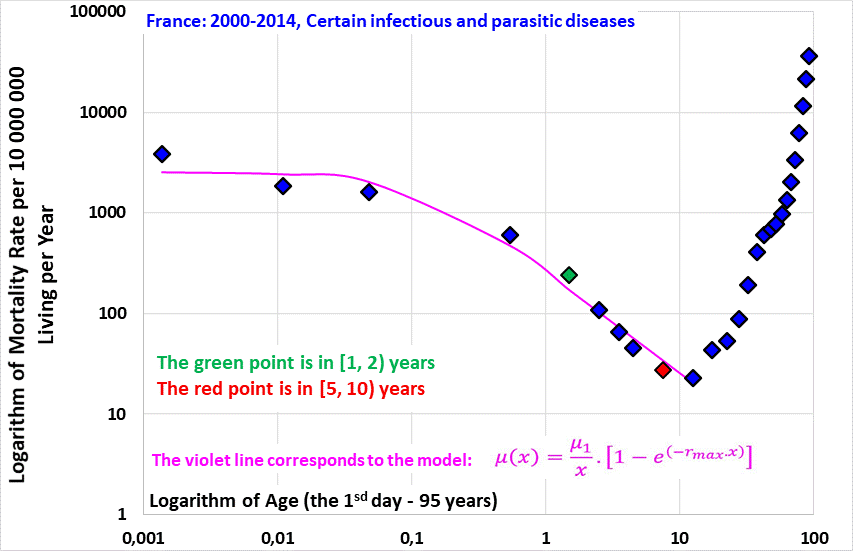

Supplement: Supplementary file 11 [file Data_Sheet_2.zip › All_Populations_Chapter_1_Animation_4.gif]

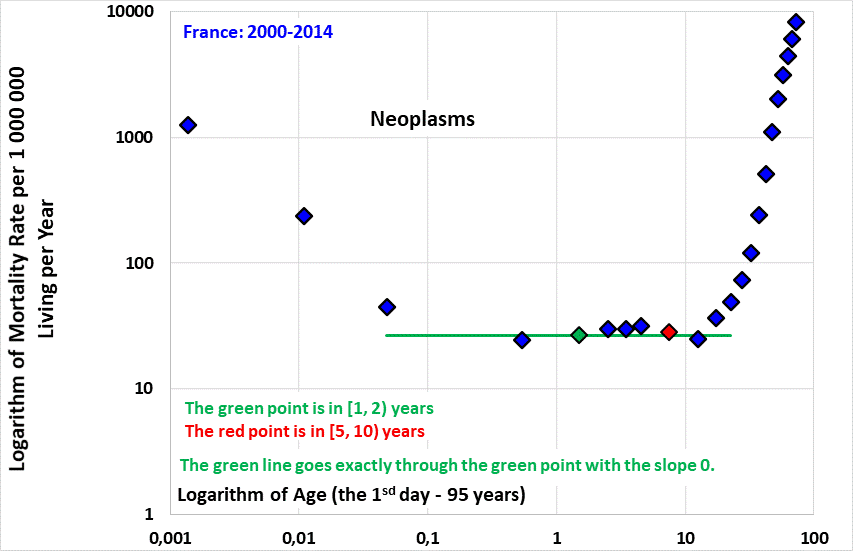

Supplement: Supplementary file 11 [file Data_Sheet_2.zip › All_Populations_Neoplasms_Animation_6.gif]

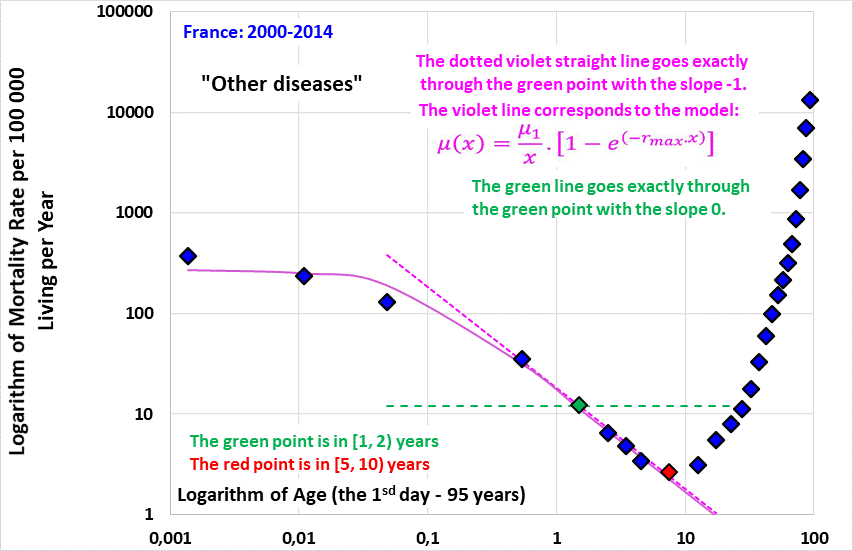

Supplement: Supplementary file 11 [file Data_Sheet_2.zip › All_Populations_Other_diseases_Animation_3.gif]
